# Correlating Physicochemical Properties of Boronic Acid-Chitosan Conjugates to Glucose Adsorption Sensitivity

**DOI:** 10.3390/pharmaceutics5010069

**Published:** 2012-12-27

**Authors:** Yaa Asantewaa, Jonathan Aylott, Jonathan C. Burley, Nashiru Billa, Clive J. Roberts

**Affiliations:** 1 School of Pharmacy, The University of Nottingham, Malaysia Campus, Jalan Broga Semenyih 43500, Selangor Malaysia; E-Mail: khyx1yaa@nottingham.edu.my; 2 School of Pharmacy, The University of Nottingham, University Park Campus, NG7 2RD, UK; E-Mails: jon.aylott@nottingham.ac.uk (J.A.); jonathan.burley@nottingham.ac.uk (J.C.B.); clive.roberts@nottingham.ac.uk (C.J.R.)

**Keywords:** phenyl boronic acid, chitosan, glucose, adsorption, polymer

## Abstract

Phenyl boronic acid (PBA), which is known to interact with glucose, was covalently bonded to chitosan by direct reductive *N*-alkylation of chitosan with 4-formylphenylboronic acid (4-FPBA). Evidence of PBA bonding on chitosan was assessed by FTIR, ToF-SIMS, SEM, DSC and glucose adsorption sensitivity measurements. FTIR spectra showed strong signals at 1560 and 630 cm^−1^ indicating the formation of *p*-substituted benzene. Similarly, ToF-SIMS analyses on the conjugates registered fragments of boron ion (B^−^) at 11.0 *m*/*z* whose intensity increased in proportion to 4-FPBA loading. The degree to which PBA was bonded to chitosan was related to the 4-FPBA load used in the reaction (termed F1 through to F6 with increasing 4-FPBA load). Glucose adsorption sensitivity to PBA-bonded chitosan was directly related to the amount of PBA functionality within the conjugates and the physical nature of the matrices (porous or crystalline). Topographic analysis by SEM revealed that PBA-chitosan conjugates F1, F2 and F3 have porous matrices and their sensitivity to glucose adsorption was directly proportional to the degree of PBA substitution onto chitosan. Conversely, conjugates F4, F5 and F6 appeared crystalline under SEM and glucose adsorption sensitivity decreased in proportion to amount of PBA bonded to chitosan. The crystalline nature of the conjugates was confirmed by DSC, where the exothermic event related to the melting of the bonded PBA moiety, occurred at 338 °C. Thus, decreased sensitivity to glucose adsorption by the conjugates can be ascribed to the crystallinity imparted by increased content of the bonded PBA moiety, providing an optimal loading of PBA in terms of maximizing response to glucose.

## 1. Introduction

Glucose sensing devices have several applications in science and medicine [1]. In medicine, there is growing interest in the development of artificially-regulated insulin delivery systems. Such systems would serve to alleviate some of the consequences associated with managing diabetes where patients rely on repeated subcutaneous insulin injections in order to maintain their blood glucose levels. 

“Boronic acid” (BA) refers to any alkyl or aryl group with boric acid attached and can form reversible covalent complexes with a number of cyclic diol functionalities including saccharides such as glucose to form five or six membered cyclic esters [2,3]. Thus, they have attracted considerable attention as alternative receptors to enzymes for glucose detection [4].

The interaction between BA and diols depends on a number of factors such as the pKa of the BA and diol, the pH of the medium and the nature of the buffer and solvent used [5], however, the relationships between these factors have yet to be fully established. A general trend that can be proposed is that binding affinity of diols to BA may increase as pH rises towards or above the pKa of the diol due to ionization and complex formation [5]. In reality these trends are not readily apparent [6]. Nonetheless, research on BA still has wide appeal due to the aforementioned potential applications. 

Until recently, research involving sensing of diol moieties by BA had been limited to analytical and signaling chemistry. These signaling themes are based on the fact that complexation of BA with diols produces a stable boronate anion which alters the electrochemical properties of the BA [7,8,9,10]. For example, in signal transduction involving boron, a complex formation on the boron atom leads to a change in its electrical properties. This change in electrical property is transferred to an appended fluorophore which is then detected [11]. As a result of this property, several BA sensing schemes have been proposed, including calorimetric [12], electrochemical [9] and fluorescence [13,14].

In constrast, there are fewer reports exploiting the glucose sensing capability of BA through its incorporation into responsive drug delivery systems. One of the early studies involved a novel polymer complex where PBA was coupled with poly (*N*-vinyl-2-pyrrolidone) to form poly (NVP-co-PBA). The resulting conjugate was then complexed with a second poly (vinyl alcohol) (PVA) component to form a gel [15]. Upon addition of glucose, the viscosity of the system dropped considerably due to competitive binding between poly (NVP-co-PBA) and PVA by glucose which has more conformational freedom. As a result, a number of delivery systems incorporating insulin were proposed to take advantage of the drop in viscosity transduced from the initial glucose sensing. In one system insulin is trapped within a PVA/poly (NVP-co-PBA) core, surrounded by a semi permeable polymer. A gel-sol transition is induced within the polymer when glucose enters the device and consequently leads to the release of insulin [16]. In another, insulin is encapsulated within a PVA/poly (NVP-co-PBA) shell which could potentially break up with glucose contact. Such systems draw parallels to those created using lectins whereby glucose binds to a sensor tagged polymer inducing a gel-sol transition [17]. 

A crucial consideration for the anchoring backbone/polymer for BA is its biological acceptability. In this regard, we decided on chitosan, a well-studied polymer known to be biodegradable and biocompatible [18,19,20,21]. It is a natural polymer and obtained by deacetylation of chitin, the second most abundant polysaccharide in nature after cellulose [22]. 

It comes as no surprise therefore that chitosan has been studied already in a number of delivery systems to date, some demonstrating the potential for insulin delivery [19,23]. Recently, Wu *et al.* [24] have investigated the utilization of phenyl boronic acid (PBA)-bonded chitosan nanoparticles as a vehicle for controlled insulin release. They attributed insulin release from the nanoparticles to glucose sensitivity of the PBA moiety and the molecular weight of the polymer. In the present investigation, we aimed to establish the relationship between changes in physicochemical properties of PBA-bonded chitosan and how this modification influenced the sensitivity of the conjugate to glucose adsorption.

## 2. Experimental Section

### 2.1. Materials

Low molecular weight chitosan (>78%) was purchased from Sigma Aldrich (St. Louis, MO, USA); tripolyphosphate (TPP), 4-formylphenyl boronic acid and sodium borohydride from Thermo Fischer Scientific (Bridgewater, NJ, USA); acetic acid, methanol, acetonitrile and glucose were purchased from Merck (Whitehouse, NJ, USA). All other chemicals were of reagent grade.

### 2.2. Synthesis of Chitosan-Phenylboronic Acid Conjugates

Four hundred micrograms of chitosan dissolved in 1% acetic acid solution was made to react with various quantities of 4-formylphenylboronicacid dissolved in methanol in the presence of sodium borohydride as reducing agent. The reaction was maintained at room temperature and made to run for 24 h. The resulting PBA-bonded chitosan conjugates (labeled as F1 through F6, Table 1) were separated from the reaction mixture by centrifugation and washing with methanol and then ethanol.

**Table 1:** Variation of 4-formylphenyl boronic acid used to formulate conjugates.

Conjugate	F1	F2	F3	F4	F5	F6
Chitosan (mg)	400	400	400	400	400	400
4-Formylphenyl boronicacid (mmol)	0.96	1.92	2.40	4.80	7.20	14.40
Sodium borohydride (mg)	240	240	240	240	240	240

### 2.3. Fourier Transform Infrared Analysis

IR spectra of chitosan, TPP, chitosan- phenylboronic acid conjugate were obtained using a Perkin Elmer FTIR Spectrometer (Spectrum RX 1). Lyophilized samples were gently mixed with about 250 mg of micronized KBr powder that was oven-dried at 120 °C for 4 h. The mixture was compressed into a disc at 5000 psi for 5 min using a pneumatic press and infra-red spectra collected in the range of 2000–400 cm^−1^.

### 2.4. Time of Flight Secondary Ion Mass Spectroscopy (ToF-SIMS)

The tagging of PBA onto chitosan was further ascertained using a time-of-flight secondary ion mass spectrometer (IV ION-TOF equipped with a liquid metal, Bi ion gun (LMIG), (IonTof GmbH, Munster, Germany), where boron ion (B^−^) was monitored as the secondary ion fragment from the boronic acid moiety of the conjugates.

### 2.5. Differential Scanning Calorimetry (DSC)

Differential scanning thermograms were obtained using a Mettler Tolledo DSC system. The sample pan comprised of about 5 mg of lyophilized powder whereas the reference pan was a standard aluminum pan. Both sample and reference pans were heated from 25 °C to 500 °C at a heating rate of 10 °C/min under 20 mL/min purge of nitrogen gas. All samples were run in triplicate.

### 2.6. Scanning Electron Microscopy (SEM)

After a 1:10 dilution with deionized water, a drop of freshly prepared conjugate dispersion was placed onto an SEM imaging stub and left to air-dry at room temperature. The dried conjugate was then viewed under field emission-SEM (Model Quanta 400F, FEI Company, USA) at 3 kV. 

### 2.7. Glucose Adsorption Studies

Twenty micrograms of conjugate was added to 1 mL of 2 mg/mL glucose solution buffered at pH 5.4. The mixture was occasionally shaken for 1 h after which 50 μL of the supernatant was aspirated and reacted with a hexokinase kit (POINTE Scientific INC). The amount of glucose adsorbed onto the conjugates was determined as reduced nicotidamide adenine dinucleotide (NADH) by HPLC after subtraction of the unreacted glucose from the original.

## 3. Results and Discussion

### 3.1. Fourier Transform Infrared Spectra (FTIR)

A summary of the schematic chemical reaction between 4FPBA and chitosan leading to the formation of the conjugates is as shown below:

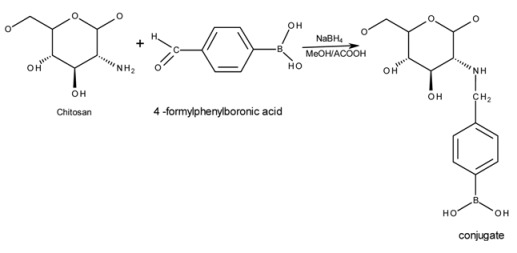



**Figure 1:**
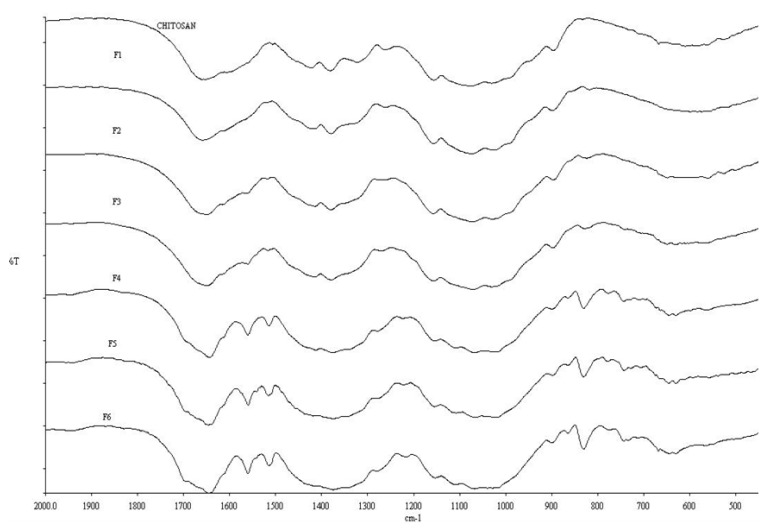
FTIR spectra of chitosan and conjugates F1 through F6 (as a function of increased PBA content).

FTIR spectra of chitosan and conjugates (Figure 1) showed the characteristic peak for chitosan at 1655 cm^−1^ attributable to an amide-linked carbonyl group, whilst the peak at 1325 cm^−1^ corresponds to a secondary amide, most likely due to incomplete de-acetylation of chitosan [25,26]. The peak relating to the primary amine of chitosan at 1590 cm^−1^ [27] became less apparent from F1 through to F6 and has virtually disappeared in conjugate F6. This strongly suggests that the PBA resides at the primary amine group (–NH_2_). The bonding of PBA on the conjugates is confirmed by the presence of peaks at 1560, 1515, 830 and 630 cm^−1^ which indicates the formation of *p*-di-substituted benzene [28]. This can only be explained if the PBA has been covalently attached to chitosan, as no such structure exists in “naked” chitosan.

### 3.2. ToF-SIMS Analysis

**Figure 2:**
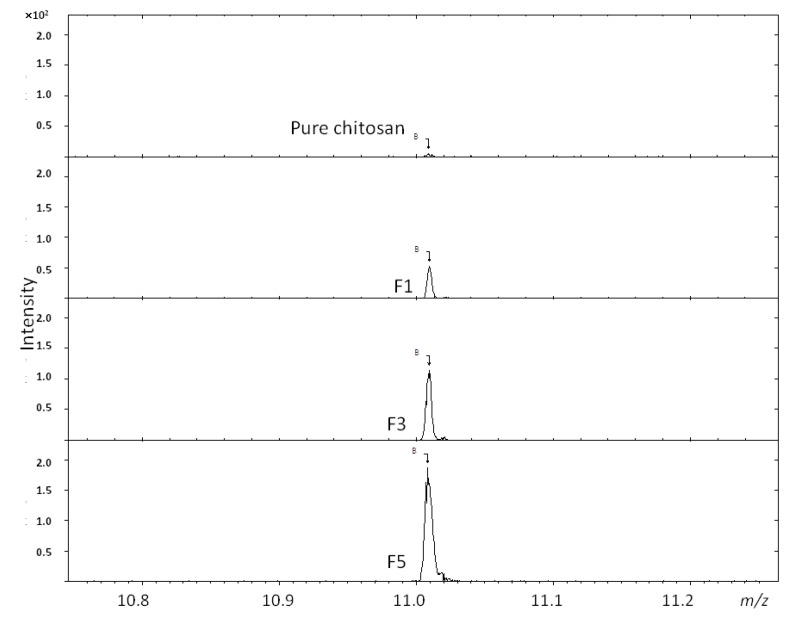
ToF-SIMS analysis of boron ion (B^−^) at 11.01 *m*/*z* from conjugates F1, F3 and F5.

The FTIR data for F1 and F2 shown in Figure 1 suggest a non-existent PBA functionality, *i.e.*, at lower 4-FPBA loading, since the peaks representing the *p*-di-substituted benzene are not observed here. The ToF-SIMS analysis, however (Figure 2), revealed the presence of boron ions (B^−^) at 11.01 *m*/*z* in F1, F3 and F5, (*i.e.*, representation of all the formulations studied). Furthermore, the intensities of these B^−^ peaks increase in proportion to the 4-FPBA load (Table 1). The B^−^ comes from tagged PBA of the conjugate since it does not appear in the pure ToF-SIMS spectrum. Thus, the ToF-SIMS analyses is in concert with the FTIR analysis in that there is a direct correlation between the degree of PBA bonding onto chitosan and the 4-FPBA loading during reaction, albeit the ToF-SIMS is more sensitive than the FTIR analysis and hence able to detect PBA functionality at lower concentration of bonded PBA within the formulations. 

### 3.3. Glucose Adsorption by Conjugates, SEM and DSC Analyses

**Figure 3:**
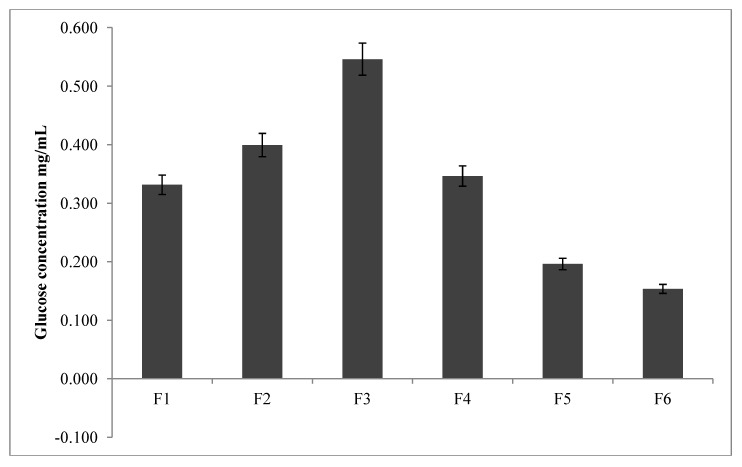
Concentration of glucose adsorbed onto conjugates as a function of boronic acid content (*n* = 3).

BA reversibly reacts with cyclic diols functionalities such as glucose to form 5- or 6-membered cyclic complexes [3]. The interaction between BA and diols may depend on the pKa of the BA and that of the diol, the pH of the medium and the nature of the buffer and solvent used [4]. Unfortunately, the interrelationships between these factors and the propensity for glucose to adsorb are poorly understood. The present study was aimed at correlating the changes in physicochemical properties of the formulated conjugates with their sensitivity to glucose adsorption (2 mg/mL). The data in Figure 3 shows that there is a direct correlation between the amount of bonded BA on the conjugates with the glucose adsorption sensitivity of F1, F2 and F3. Conversely, the sensitivity to glucose adsorption on F4, F5 and F6 reduces in that order at glucose concentration studied (F3 adsorbing the highest amount of glucose in all the formulations). 

**Figure 4:**
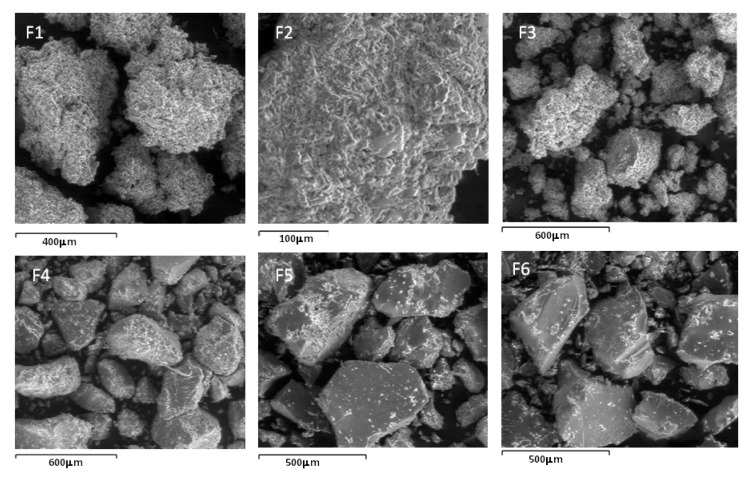
SEM images of conjugates F1 through F6.

SEM images of the conjugates presented in Figure 4 reveal a porous matrix for F1, F2 and F3 whilst those of F4, F5 and F6 appear to be crystalline. It seems plausible from the SEM images of the conjugates that due to the porous nature of F1, F2 and F3 matrices, glucose adsorption onto these conjugates proceeds unrestricted. In contrast, due to the crystalline matrices of F4, F5 and F6, adsorption by glucose is inhibited because of a close packing arrangement even though these series (F4, F5 and F6) have a higher concentration of bonded PBA compared to the former series. Nevertheless, the glucose adsorption study corroborates the FTIR/ToF-SIMS analyses for F1, F2 and F3 in that a positive correlation exists between glucose adsorption and the concentration of PBA bonded onto these conjugates. 

In a related study Wu *et al.* [24] attributed restricted swelling of PBA-chitosan nanoparticles (an indication of poor PBA-glucose interaction) at high PBA to chitosan concentration to the bidentate binding nature of glucose by two the two –OH groups within the BA moiety. In this regard, the adsorbed glucose anchors as a cross-linker and hence restricts swelling of the PBA-chitosan nanoparticles which impedes further glucose adsorption due to inaccessibility of glucose to further adsorption by PBA. This effect of reduced PBA-chitosan swelling due to adsorbed glucose was enhanced when the concentration of PBA was high. In the present study, the SEM investigations point to the formation of crystalline matrices in F4, F5 and F6, (*i.e*., at higher PBA-chitosan concentration). It appears that the change from porous (F1, F2, F3) to crystalline conformities accounts for the decreased sensitivity to glucose adsorption.

**Figure 5:**
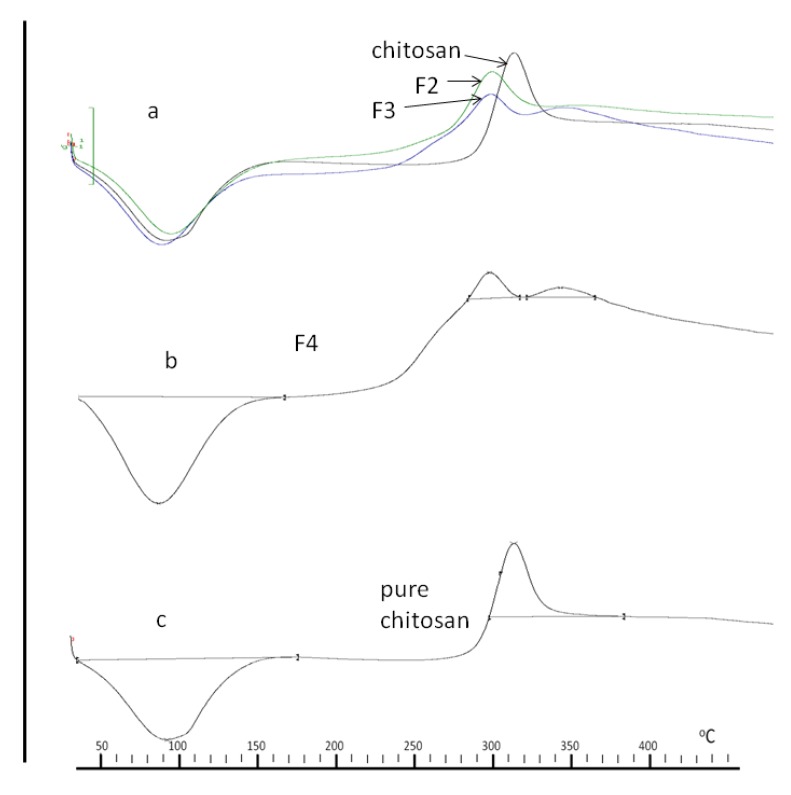
DSC thermograms of chitosan and conjugates (**a**) F2, F3 and chitosan, (**b**) F4 and (**c**) chitosan.

To study the change in material organisation in chitosan due to bonding with PBA, DSC analyses of the conjugates were performed and are presented in Figure 5 as (a) F2, F3 & pure chitosan, (b) F4, (c) pure chitosan. Due to their facile dehydration on heating, pure PBA crystals tend to give inconsistent melting temperatures; thus, true melting points are reflective of decomposition temperatures [29]. In all of the thermograms, an endothermic peak was manifested at 86.4 °C and is due to the liberation of water from the –NH_2_ and –OH groups within the chitosan backbone [30]. An exothermic peak from pure chitosan (Figure 5c) occurs at 308.3 °C and represents decomposition of –NH_2_ groups. This decomposition of –NH_2_ groups also occurs in conjugates F2, F3 and F4 (Figure 5a,c), albeit at a lower temperature (293.2 °C). A reduction in decomposition temperature of chitosan is due to consumption of –NH_2_ groups by bonded the moiety [31] and in the present study, attests to the bonding of PBA to chitosan. 

All three conjugates registered exothermic peaks at 338.5 °C which is absent in the pure chitosan thermogram (Figure 5c), therefore we can ascribe this exothermic peak to bonded PBA, which interestingly, increases in intensity with increases in PBA to chitosan concentration. We can conclude that the exothermic peak at 338.5 °C is related to the melting enthalpy of the bonded PBA moiety within the conjugates which is also responsible for imparting crystallinity to the conjugates. This conclusion is supported by a similar observation with chitosan-poly vinyl alcohol (PVA) conjugates where it was reported that the degree of crystallinity of the conjugates decreased with increase in chitosan content, given that the crystallinity of the conjugates was due to PVA [32].

## 4. Conclusions

PBA-bonded chitosan conjugates were successfully synthesized. We have also demonstrated that the degree of PBA substitution can be altered in a controlled and predictable fashion by varying the 4-FPBA loading at reaction. The bonding of PBA to chitosan was via the –NH_2_ groups of chitosan which became consumed with higher 4-FPBA loadings. This was evident through a reduced decomposition temperature. The presence of PBA also modified the appearance of the conjugates from porous matrices to crystalline ones with increasing PBA bonding. The crystalline conjugates were less sensitive to glucose adsorption due to inaccessibility of glucose to the PBA moiety. Thus, sensitivity to glucose adsorption by the conjugates appears to require an optimum content of boronic acid, beyond which crystalline domains form which reduces access to glucose. These results have clear implications for the development of glucose sensing and in particular for delivery systems based upon boronic acid-tagged chitosan.
